# IK channel activation increases tumor growth and induces differential behavioral responses in two breast epithelial cell lines

**DOI:** 10.18632/oncotarget.16389

**Published:** 2017-03-21

**Authors:** Amy E. Thurber, Michaela Nelson, Crystal L. Frost, Michael Levin, William J. Brackenbury, David L. Kaplan

**Affiliations:** ^1^ Program in Cell, Molecular, and Developmental Biology, Sackler School of Graduate Biomedical Sciences, Tufts University, Boston, Massachusetts, USA; ^2^ Department of Biomedical Engineering, Tufts University, Medford, Massachusetts, USA; ^3^ Department of Biology, University of York, Heslington, York, UK; ^4^ Biology Department, and Tufts Center for Regenerative and Developmental Biology, Tufts University, Medford, Massachusetts, USA

**Keywords:** oncochannel, bioelectricity, potassium channel, cancer

## Abstract

Many potassium channel families are over-expressed in cancer, but their mechanistic role in disease progression is poorly understood. Potassium channels modulate membrane potential (V_mem_) and thereby influence calcium ion dynamics and other voltage-sensitive signaling mechanisms, potentially acting as transcriptional regulators. This study investigated the differential response to over-expression and activation of a cancer-associated potassium channel, the intermediate conductance calcium-activated potassium channel (IK), on aggressive behaviors in mammary epithelial and breast cancer cell lines. IK was over-expressed in the highly metastatic breast cancer cell line MDA-MB-231 and the spontaneously immortalized breast epithelial cell line MCF-10A, and the effect on cancer-associated behaviors was assessed. IK over-expression increased primary tumor growth and metastasis of MDA-MB-231 in orthotopic xenografts, demonstrating for the first time in any cancer type that increased IK is sufficient to promote cancer aggression. The primary tumors had similar vascularization as determined by CD31 staining and similar histological characteristics. Interestingly, despite the increased *in vivo* growth and metastasis, neither IK over-expression nor activation with agonist had a significant effect on MDA-MB-231 proliferation, invasion, or migration *in vitro*. In contrast, IK decreased MCF-10A proliferation and invasion through Matrigel but had no effect on migration in a scratch-wound assay. We conclude that IK activity is sufficient to promote cell aggression *in vivo*. Our data provide novel evidence supporting IK and downstream signaling networks as potential targets for cancer therapies.

## INTRODUCTION

Breast cancer is one of the most common forms of cancer with roughly 230,000 new cases per year and is responsible for 40,000 deaths in the United States alone. About 1 in every 8 women will be diagnosed with breast cancer at some point in her lifetime. Women diagnosed with localized breast cancer have an excellent prognosis with over 98% 5 year survival. Unfortunately, patients with metastases have only a 25.9% 5 year survival highlighting the need for treatments aimed at metastasis [1]. A major hindrance to the development of better breast cancer treatments is a lack of understanding of what drives disease progression to metastasis. For decades it has been known that some cancers remain in a benign state for long time periods while others quickly progress to malignancy, and that generally only a subset of tumor cells are capable of metastasis. Although many metastasis-specific pathways have been described, our understanding of the upstream signals that activate these pathways, and the factors that cause certain cells to metastasize while nearby syngeneic cells do not, remain largely unknown [2].

Over the past decade ion channels have been receiving increased attention for their roles in cancer progression. Numerous cancer types have altered ion channel expression, with expression of certain channels correlating with cancer stage [3]. In various cases, inhibiting these channels leads to a decrease in cancer-associated behaviors including primary tumor growth and metastasis *in vivo* [4–6]. Most studies have focused on ion channels as downstream targets of signaling pathways that execute critical mechanical functions required for aggressive behaviors. For instance, inhibiting certain chloride and potassium channels responsible for generating changes in cell volume decreases cell migration and proliferation [7]. However, evidence suggests ion channels may have upstream regulatory roles as well, and little is known about the ability of ion channel activity to initiate signaling cascades to promote aggressive cancer behaviors [8, 9].

The intermediate conductance calcium-activated potassium channel (IK) is over-expressed in numerous cancer types including breast, prostate, uterus, stomach, colorectal, pancreas, pituitary gland, and brain cancers [10] and inhibiting IK decreases cancer cell proliferation, migration, and *in vivo* tumor growth and metastasis [11–16]. Based on these results, the widely held theory in the field is that IK is a downstream effector of signaling pathways and is required in the late steps of enacting aggressive cancer behaviors. However, IK may have additional upstream instructive roles and its activity may be sufficient to initiate aggressive behaviors through its effect on calcium dynamics. In prostate cancer cells, activation of IK with its agonist was sufficient to significantly increase intracellular calcium concentrations suggesting IK could regulate downstream calcium-dependent signaling pathways [17]. Furthermore, IK activation was sufficient to increase prostate cancer proliferation, providing additional evidence of the ability of IK to activate signaling pathways [12]. However, the possible sufficiency of IK to promote aggressiveness has not been previously studied in breast cancer cells.

In the present study, our aims were (1) to investigate whether increased IK activity was sufficient to promote proliferation in breast epithelial cells and cancer cells and (2) to investigate whether an increase in IK was also sufficient to increase other aggressive cancer behaviors, including tumor growth and metastasis *in vivo*. We chose to use the metastatic breast cancer cell line MDA-MB-231 because IK inhibition studies demonstrated decreased proliferation, migration, and colony formation indicating IK has an important physiological role in aggressive behaviors in this cell line [18]. Surprisingly, we found that MDA-MB-231 *in vitro* proliferation, invasion, and migration were not affected by IK over-expression or activation. Interestingly, however, increased IK decreased proliferation and invasion of the spontaneously immortalized breast epithelial non-tumorigenic MCF-10A cell line but had no effect on migration. In contrast to the *in vitro* results, we found that over-expressing IK in MDA-MB-231 was sufficient to increase primary tumor growth and metastasis in mice. This study is the first to demonstrate the sufficiency of IK to increase cancer aggression *in vivo* and suggests the possibility of key differences in behavioral response to IK activation between tumorigenic and non-tumorigenic cells, although more cell lines must be tested to determine a potential trend. Our results indicate that IK plays an important instructive role in cancer progression and suggest the possibility of unique signaling mechanisms that could be used as specific targets.

## RESULTS

### IK over-expression increases potassium current and hyperpolarizes V_mem_

**Figure 1:**
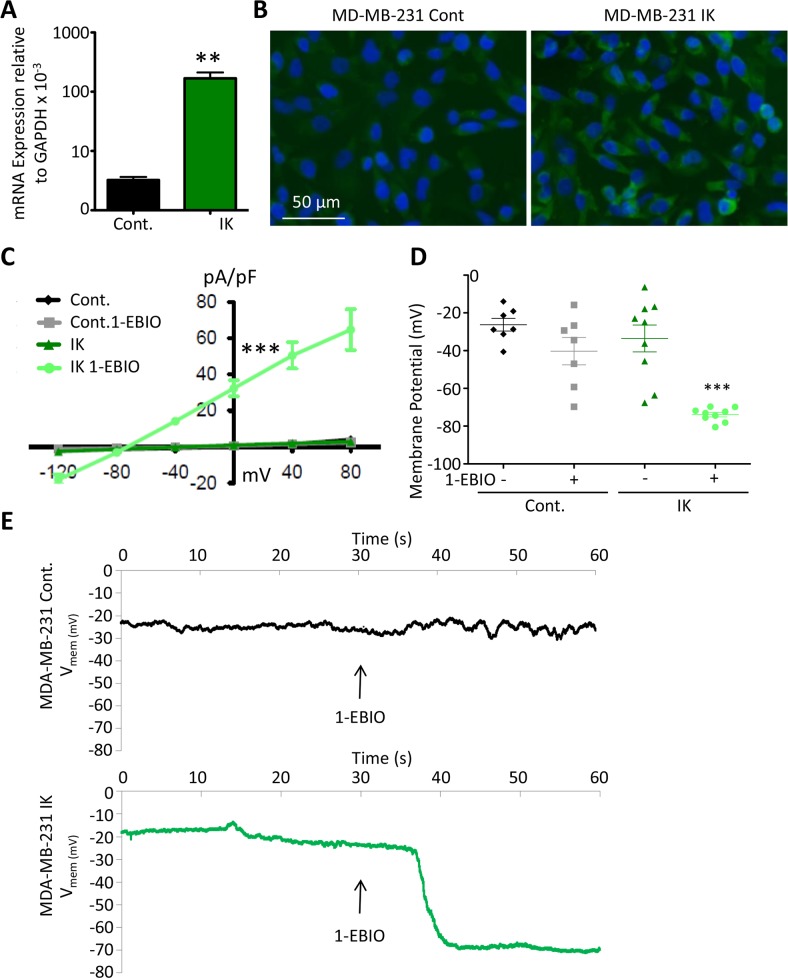
Functional contribution of IK over-expression to current density and V_mem_ **(A)** IK mRNA expression levels relative to GAPDH in total RNA collected from MDA-MB-231 infected with pMIG-RFP (Cont.) or pMIG-IK (IK) and selected for RFP fluorescence by FACS. Data are presented as mean with standard error of the mean (SEM) of 3 independent replicates (** significant difference p < 0.01, 2 sample t-test). **(B)** MDA-MB-231 control or MDA-MB-231-IK were fixed on coverslips and immunofluorescence microscopy was performed using antibodies to IK (green) with DAPI (blue) staining of the nuclei. Increased intensity of the green IK staining is evident in the MDA-MB-231-IK sample. **(C)** Endogenous and 1-EBIO induced current-voltage relationship in control and IK-expressing cells recorded in the cell attached perforated patch configuration from MDA-MB-231 cells. Data are presented as mean ± SEM from a minimum of 7 recordings. The current density was significantly increased in MDA-MB-231-IK 1-EBIO treated cells as compared to control vehicle treated, control 1-EBIO treated, and MDA-MB-231-IK vehicle treated cells (*** p < 0.001, 1-way ANOVA of 0 mV current density) **(D)** V_mem_ averaged over 20 seconds from recordings of same cells as C. Data points represent individual cells, bars show mean with SEM (*** indicates significantly different than vehicle treated control p < .001, 1-way ANOVA). **(E)** V_mem_ recording of representative MDA-MB-231 control cell (top) and MDA-MB-231-IK cell (bottom) with bath solution exchanged to 1-EBIO solution beginning at 30 seconds and continuing through duration of recording. There is a ~7 second delay between when the solutions are changed and when the new solution reaches the cells.

In order to test the sufficiency of increased IK to induce increased aggression in the breast cancer cell line MDA-MB-231, we first generated cells with increased IK expression. Cells were infected by a retrovirus encoding either IK and red fluorescent protein (RFP) or RFP alone as vector control and selected for RFP using fluorescence activated cell sorting (FACS) (Supplementary Figure 1). MDA MB 231 have previously been reported to endogenously express IK [19](data accessible at NCBI Geo database GSE41678). We confirmed that IK was expressed in control cells (MDA-MB-231-RFP) by RT-PCR and that cells infected with IK virus (MDA-MB-231-IK) had significantly increased IK expression (p = 0.0027, 2-sample t-test, Figure 1A). Overexpression was further confirmed at the protein level by immunofluorescence (Figure 1B).

To validate functional activity of the over-expressed IK channel, the electric current density and V_mem_ of individual cells were measured in the presence or absence of 200 μM of the IK agonist 1- Ethylbenzimidazolinone (1-EBIO). It should be noted that 1-EBIO is also an agonist of the closely related small conductance calcium activated potassium channels (SK) [20]. However, SK transcripts were below the limit of detection using RT-PCR suggesting that IK is more prevalent. Furthermore, treatment of MDA-MB-231 with the IK specific antagonist TRAM-34 inhibits most calcium-sensitive potassium current indicating that SK has lower endogenous activity [18]. Electrophysiological recordings were acquired in the cell attached configuration using a perforated patch in order to best maintain the endogenous cytosolic contents and preserve V_mem_. 1-EBIO treatment had no effect on current density of control MDA-MB-231-RFP cells (q = 0.15; 1-way ANOVA of 0 mV current density). Similarly, IK over-expression had no effect on current density in the absence of 1-EBIO (q = .31; 1-way ANOVA of 0 mV current density). However, in MDA-MB-231-IK cells, 1-EBIO treatment significantly increased the current density from 0.98 ± 2.32 pA/pF to 32.33 ± 13.60 pA/pF (p < .001, q = 12.30 1-way ANOVA of 0 mV current density; Figure 1C). Furthermore, the current density of MDA-MB-231-IK treated with 1-EBIO was significantly larger than either untreated or 1-EBIO treated control cells (q = 11.31 and q = 11.14 respectively, 1-way ANOVA of 0 mV current density). The reversal potential for MDA-MB-231-IK EBIO treated cells was -76.21 ± 2.58 mV, relatively near the potassium reversal potential predicted by the Nernst equation (−89.9 mV), indicating the current was comprised primarily of potassium flux. These data suggest that MDA-MB-231-IK cells express significantly more functional IK channels than control MDA-MB-231-RFP cells.

IK over-expression without agonist treatment did not significantly alter the V_mem_ of MDA-MB-231 cells (control -26.3 ± 8.2 mV, MDA-MB-231-IK -33.5 ± 16.5 mV, q = 1.34, 1-way ANOVA). However, there was a greater range of V_mem_ values in the MDA-MB-231-IK population (range of -6.4 to -67.6 mV) compared to control cells (range of -13.9 to -40.6 mV; p = 0.045; F test to compare variance) with the IK population being skewed towards hyperpolarized (control -0.26, MDA-MB-231-IK -0.62, adjusted Fisher-Pearson standardized moment coefficient), suggesting IK over-expression leads to increased basal levels of conductance in some cells or is transiently active (Figure 1D). Application of 1-EBIO significantly hyperpolarized the V_mem_ of MDA-MB-231-IK cells to -73.9 ± -2.8 mV (p < .001, paired 2-sample t-test). 1-EBIO also hyperpolarized the V_mem_ in control cells, although this was not statistically significant (−40.3 ± 17.7 mV; p = 0.06 paired 2-sample t-test). Additionally, control cells exhibited a fluctuation in V_mem_ at the time of 1-EBIO application, suggesting the presence of active IK or SK channels (Figure 1E).

### MDA-MB-231 *in vitro* proliferation, invasion, and migration are unaffected by IK over-expression and activation

We next investigated the effect of IK expression and activity on *in vitro* behaviors of MDA-MB-231 cells. IK is endogenously activated by calcium signaling and its activation is highly temporally and spatially regulated which may be important to its downstream function [13]. We wanted to compare the effect of increased IK current responding to endogenous temporal and spatial activation as well as to low and high levels of continuous pan-IK activation. Cells over-expressing IK were expected to maintain endogenous activation patterning but with larger currents. We therefore compared the behavioral response of control and IK over-expressing cells treated or untreated with agonist.

**Figure 2:**
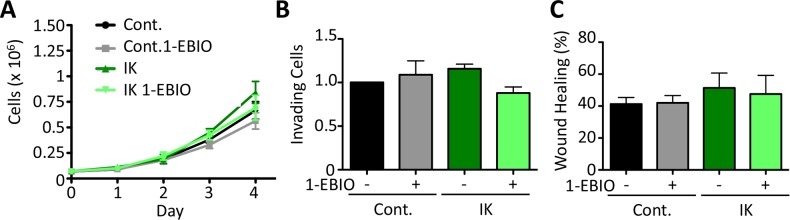
IK over-expression and activation with 1-EBIO had no effect on MDA-MB-231 proliferation, invasion, or migration **(A)** Proliferation of control and IK over-expressing MDA-MB-231 cells was quantified over 4 days for cells treated with vehicle control or 1-EBIO. There was no significant difference in cell number on any day (Day 4: p = 0.33, 1-way ANOVA). **(B)** Invasion through Matrigel coated transwell after 16 h using FBS as a chemoattractant. Average number of cells per field of view with 4 fields of view per sample and 4 replicates in each of 3 independent experiments. There was no significant difference in the number of invading cells across any condition (p = 0.32, 1-way ANOVA) **(C)** A confluent layer of MDA-MB-231 control or MDA-MB-231-IK was scratched and healing was assessed after 10 h with vehicle control or 1-EBIO treatment. There was no significant difference in the normalized surface area of wound recovery (p = 0.41, 1-way ANOVA). All data presented as mean with SEM of three independent replicates.

It has been previously reported that IK over-activation by agonist treatment increases proliferation in prostate cancer cells [12]. Here, we monitored the proliferation of both control and IK over-expressing MDA-MB-231 cells over 4 days in the presence or absence of 1-EBIO. We found that there was no significant difference in proliferation across any of the conditions (Day 4: p = 0.33 1-way ANOVA; Figure 2A). We next evaluated *in vitro* behaviors associated with metastasis. The ability of cells to invade was assayed *in vitro* using a transwell assay in which the number of cells invading across a Matrigel-coated membrane in response to a serum gradient was quantified. There was no significant difference in the number of invading cells across any of the conditions (p = 0.32 1-way ANOVA; Figure 2B). Similarly, migration in a scratch wound assay was not altered, with no significant difference in the surface area of wound recovery after 10 h (p = 0.41 1-way ANOVA; Figure 2C). These results suggest that increased IK activity is not sufficient to increase *in vitro* measures of MDA-MB-231 cell aggression.

### IK over-expression and activation decreases colony formation of MDA-MB-231 Cells

**Figure 3:**
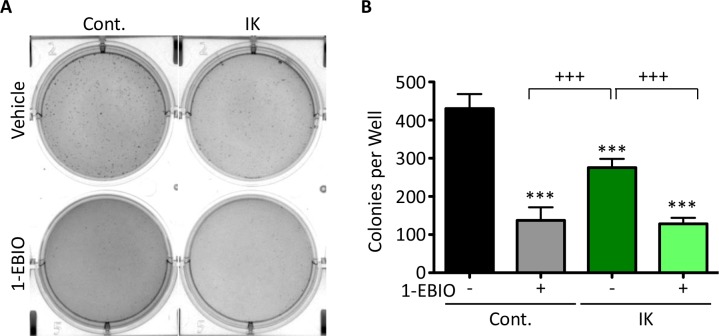
MDA-MB-231 soft agarose colony formation is decreased by both IK expression and IK activation **(A)** Bright field images of crystal violet stained MDA-MB-231 control and MDA-MB-231-IK cells grown in soft agarose and treated with vehicle control or 1-EBIO for 28 days. **(B)** Quantification of colonies from A. Data presented as mean with standard deviation of three independent replicates, *** indicates significantly different than vehicle treated control, +++ significant difference between indicated samples (p < 0.001 1-way ANOVA).

The ability to form colonies in soft agar is used as an *in vitro* measure of transformation and tumor forming ability. Inducing expression of oncogenes such as c-MYC or RAS in non-tumorigenic cell lines can confer colony forming ability which correlates with tumor formation in mouse xenografts demonstrating the capacity of this assay to assess conversion to a malignant phenotype [21]. MDA-MB-231 cells are highly malignant and therefore readily form colonies. Given that IK is over-expressed in many metastatic cancers, we anticipated increased IK activity would increase colony formation. Surprisingly, colony formation in IK-expressing cells was decreased to 64% as compared to untreated control and was further decreased to 30% by 1-EBIO treatment (Figure 3A–3B; p < 0.001 1-way ANOVA). 1-EBIO similarly reduced the colony formation of control MDA-MB-231 cells to 32% of untreated cells. These data suggest that treatment with 1-EBIO, which induces constant IK activation, more severely inhibits colony formation than elevated transiently activated IK induced by increased IK expression.

### IK activation decreases MCF-10A proliferation and invasion

MDA-MB-231 cells are highly aggressive and so the lack of effect of IK expression/activity on cell proliferation, migration, and invasion may be due to an inability to further augment these already aggressive behaviors. We therefore tested the effect of increased IK expression and activation in the non-tumorigenic breast epithelial MCF-10A cell line. We created an IK over-expressing population of MCF-10A cells. As with MDA-MB-231, MCF-10A-IK cells had dramatically increased IK expression (p < 0.001, 2-sample t-test; Supplementary Figure 2A) and increased current density at 0 mV with 1-EBIO treatment from 0.483 +/− 0.344 pA/pF in control cells to 22.90 +/− 18.46 pA/pF in MCF-10A-IK (p < .001, q = 7.30 1-way ANOVA, Supplementary Figure 2B). IK over-expression without agonist treatment hyperpolarized the V_mem_ of MCF-10A cells from -21 ± 6.3 mV in control cells to -47 ± 14.7 mV in MCF-10A-IK cells (p < 0.001, 1-way ANOVA; Supplementary Figure 2C) indicating the overexpressed channels were activated by endogenous signaling. Application of 1-EBIO hyperpolarized MCF-10A control cell V_mem_ to -31 ± 7.4 mV (p < 0.01, paired t test) indicating that the cells have endogenous IK or SK channels. 1-EBIO treatment induced a larger effect on MCF-10A-IK cells, hyperpolarizing the V_mem_ to -69 ± 6.9 mV (p < 0.01, paired t-test). Although the effect of 1-EBIO treatment may have been partially mediated by SK channel activation, we were not able to detect SK transcripts by RT-PCR suggesting SK has either very low or no expression in MCF-10A cells. In summary, these data demonstrate that IK overexpression in MCF-10A cells induces a similar change in electrical properties to MDA-MB-231 cells.

**Figure 4:**
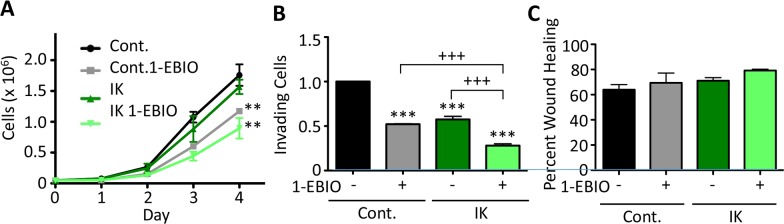
1-EBIO treatment decreased MCF-10A proliferation and invasion but had no effect on migration **(A)** Control and IK over-expressing MCF-10A proliferation was quantified over 4 days for cells treated with vehicle control or 1-EBIO (** p < 0.01 indicates significantly different than vehicle control, 1-way ANOVA). **(B)** Invasion through Matrigel-coated transwell after 24 h using horse serum and EGF as a chemoattractant. Average number of cells per field of view with 4 fields of view per sample and 4 replicates in each of 3 independent experiments (*** p < 0.001 indicates significantly different than vehicle treated control, +++ p < 0.001 indicates significant difference between indicated samples, 1-way ANOVA). **(C)** A confluent layer of MCF-10A control or MCF-10A-IK was scratched and treated with vehicle control or 1-EBIO. Healing was assessed after 12 h by quantifying the surface area of wound recovery (no significant difference across all conditions; p = 0.28, 1-way ANOVA). All data presented as mean with SEM of three independent replicates.

We next studied the effect of IK expression and activity on *in vitro* measures of aggression in MCF-10A cells. IK over-expression alone had no effect on MCF-10A cell proliferation, but activation of IK by 1-EBIO treatment significantly decreased proliferation of control MCF-10A cells by 33 ± 4 % and IK expressing cells by 43 ± 16 % (p < 0.01, 1-way ANOVA; Figure 4A). MCF-10A invasion was significantly decreased by both IK over-expression and activation (p < 0.001, 1-way ANOVA; Figure 4B). It should be noted that in the control condition, less than 1% of the cells loaded in the upper chamber invaded to the lower chamber reflecting similar basal invasion levels as has been reported elsewhere and significantly reduced as compared to MDA-MB-231 [22–24]. However, no significant change in cell migration using a wound scratch assay was observed across any condition (p = 0.28, 1-way ANOVA; Figure 4C). Finally, we tested the effect of IK expression and activity on colony-forming ability. MCF-10A is an immortal but not transformed cell line and is unable to form colonies in soft agarose but can be induced to form colonies by expression of strong oncogenes [25]. Neither MCF-10A control nor MCF-10A-IK cells were able to form colonies with or without 1-EBIO treatment (Supplementary Figure 3).

### IK over-activation by 1-EBIO causes MCF-10A but not MDA-MB-231 cells to accumulate in G2

**Figure 5:**
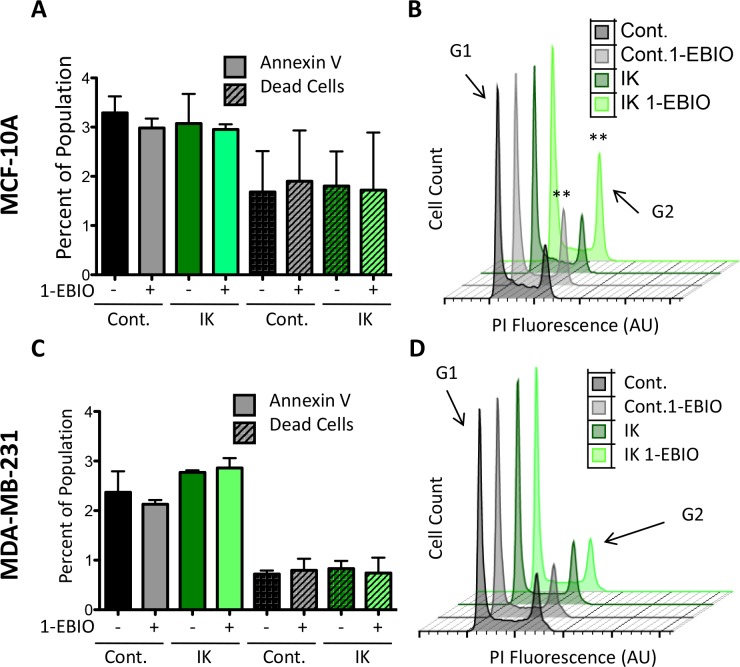
1-EBIO treatment increased G2 phase accumulation in MCF-10A but had no effect on MDA-MB-231 Control and IK over-expressing MCF-10A **(A)** and MDA-MB-231 **(C)** cells were treated with vehicle control or 1-EBIO for 24 h, stained with annexin-FITC and violet dead stain, and analyzed by flow cytometry to quantify the percentage of positively stained cells (no significant difference in annexin-V or dead stained cells across any condition, 1-way ANOVA). **(B, D)** Plot of cell count versus propidium iodide (PI) fluorescence to analyze cell cycle. Arrow marks fluorescence intensity corresponding to G2 phase. Plot is representative of three independent replicates (MCF-10A **p < 0.01, MDA-MB-231 p = 0.29, 1-way ANOVA). All data presented as mean with SEM of three independent replicates.

We initially hypothesized that the differential proliferative response between MCF-10A and MDA-MB-231 cells was due to a difference in apoptosis sensitivity. High potassium currents have been reported to induce apoptosis and one possibility was that MCF-10A cells are sensitive to large potassium currents while MDA-MB-231 cells could be protected by anti-apoptotic signaling that is often active in highly metastatic cells [26]. To test this, we measured the percentage of apoptotic and dead cells to determine if the decrease in MCF-10A cell number following IK activation was due to an increase in cell death. Control and IK over-expressing cells were cultured for 24 hours in 1-EBIO or vehicle control and stained with Annexin-V-FITC, which binds external phosphatidylserine an early marker of apoptosis, and violet dead cell stain. FACS was used to quantify the percentage of labeled cells. There was no significant difference in the percentage of apoptotic or dead cells across all cell conditions for both MCF-10A and MDA-MB-231 cells (Figure 5A, 5C). We next investigated changes in the cell cycle distribution in the same samples using propidium iodide staining. 1-EBIO treatment increased the percentage of control and IK-overexpressing MCF-10A cells in the G2 phase (from 14.80 ± 3.71 % to 18.48 ± 3.27 % in control MCF-10A cells and from 16.08 ± 2.18 % to 21.4 ± 5.1 % in MCF-10A-IK cells; p < 0.01, 1-way ANOVA; Figure 5C). However, no significant differences were found in any of the conditions for MDA-MB-231 cells (p = 0.29, 1-way ANOVA; Figure 5D). The increased G2 accumulation was therefore observed in the same populations that had decreased proliferation. These data suggest that in MCF-10A cells the G2/M checkpoint includes a requirement for decreased IK activity and/or depolarization and that MDA-MB-231 cells are able to evade this requirement, possibly due to strong pro-growth signaling active in this cell line.

### IK expression increases MDA-MB-231 primary tumor growth and metastasis

**Figure 6:**
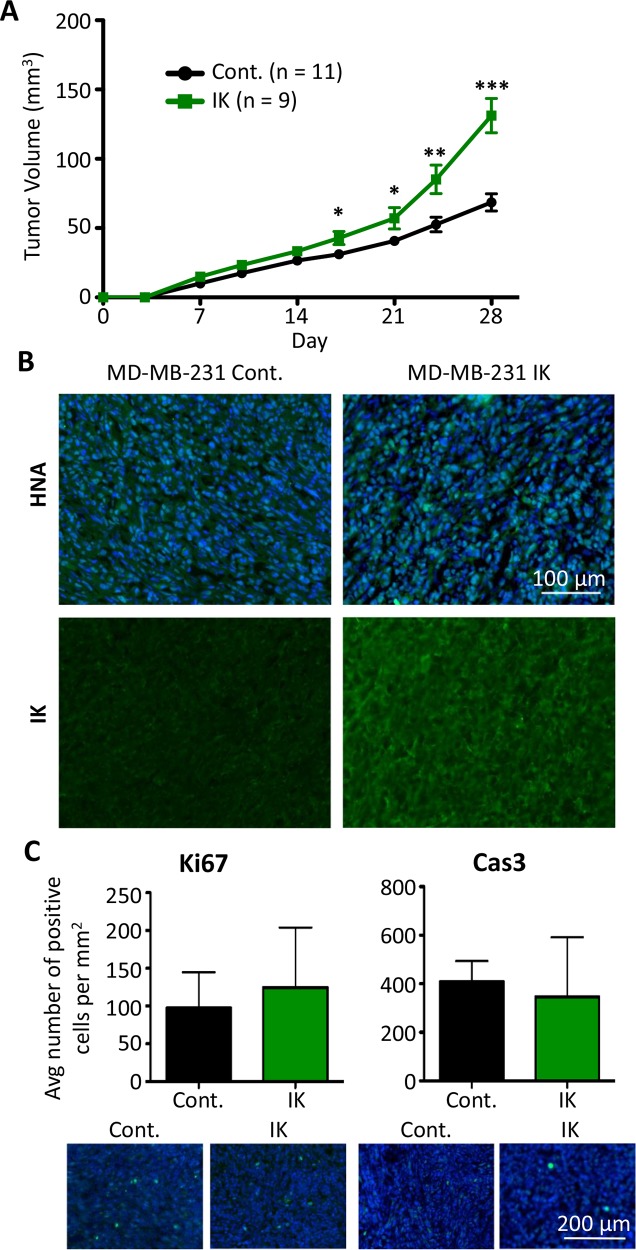
IK over-expression increases *in vivo* tumor growth of MDA-MB-231 **(A)** MDA-MB-231 control (n = 11) and MDA-MB-231-IK (n = 9) cells were injected into the inguinal mammary fat pad of 6 wk old female mice and tumor size was plotted over time. Data presented as mean with SEM (* p < .05, ** p < .01, ***p < .001 2 sample t-test). **(B)** Fixed primary tumor sections stained with antibody to human nuclear antigen (green) with DAPI (blue) staining of the nuclei (top) or with anti-IK antibody (green; bottom). **(C)** Quantification of Ki67 positive (left) and active Caspase-3 positive (right) cells in primary tumor sections with representative images of Ki67 and active caspase-3 (green) staining with DAPI (blue) staining of the nuclei shown below. There was no significant difference in the percentage of either Ki67 or active caspase-3 positive cells (p = 0.27, 2-sample t-test and p = 0.15, 2-sample t-test).

Given the various effects of IK expression and activity on cellular aggressiveness *in vitro*, we next assessed the effect of IK over-expression on *in vivo* tumor growth and metastasis. MDA-MB-231 control and MDA-MB-231-IK cells were injected into the mammary fat pad of 6 wk old female *Rag2^−/−^ Il2rg^−/−^* mice. Caliper measurements were used to estimate the tumor volume over 4 wks of tumor growth. Primary tumor growth was significantly increased in MDA-MB-231-IK tumors beginning 17 days post cell injection (p < 0.05, 2-sample t test; Figure 6A). To demonstrate that the tumors were derived from the injected cells, tumor sections were stained for human nuclear antigen (HNA) and cells stained positively throughout the bulk of the tumor (Figure 6B). MDA-MB-231-IK tumors showed higher IK immunoreactivity compared to control tumors, suggesting that IK over-expression was retained *in vivo* (Figure 6B). Despite the increase in tumor size, there was no significant difference in the number of Ki67 positive proliferating cells in 28 day tumor sections (p = 0.27, 2-sample t-test; Figure 6C). There was also no significant difference in the number of cleaved caspase-3 positive apoptotic cells in tumor sections at day 28 (p = 0.15, 2-sample t-test; Figure 6E). However, there was a trend towards increased Ki67+ cells and reduced caspase-3+ cells in the IK-overexpressing tumors, suggesting that increased proliferation and/or reduced apoptosis may contribute to the increased size of these tumors. In addition, as these measurements were taken at the end of the experiment, they may underestimate a larger difference in proliferation or apoptosis that occurred earlier on in the growth of the tumor.

**Figure 7:**
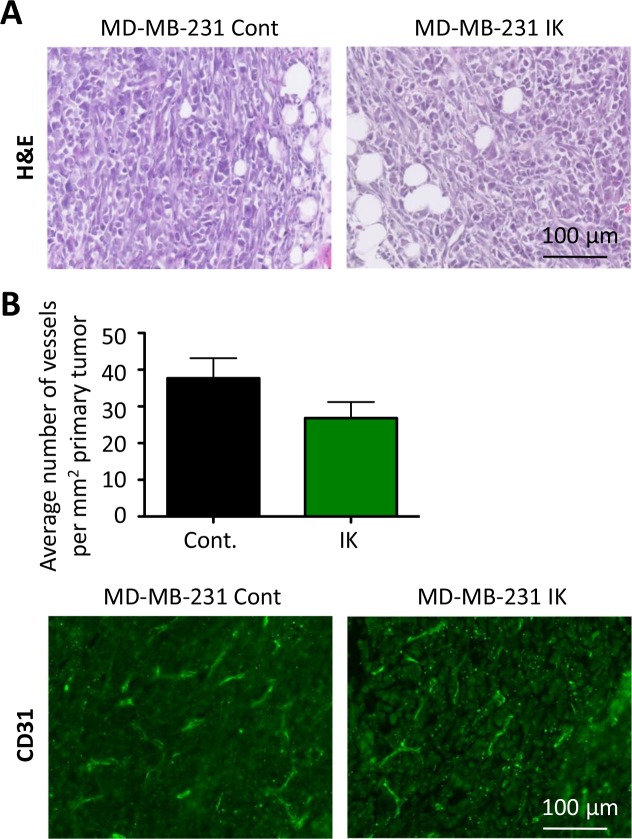
No evidence of increased aggression in primary tumor **(A)** Primary tumor sections stained with H&E. **(B)** Quantification of vessel density in primary tumors (top) with representative CD31 stained sections (bottom, green - CD31, blue - nuclei stained with DAPI; p =0.14, 2-sample t-test).

We next investigated whether the primary tumors also displayed differences in aggressive phenotypes. Staining of primary tumor sections with H&E revealed similar local invasion into surrounding muscle and fibroadipose tissue from both MDA-MB-231 control and MDA-MB-231-IK primary tumors (Figure 7A). To assess vascular density, primary tumor sections were stained with the endothelial marker CD-31 and the number of vessels per square millimeter was quantified. There was no significant difference in the vascular density between control and MDA-MB-231-IK tumors and surprisingly if anything there was a trend towards denser vascularization in control cells (p = 0.14, 2-sample t-test, Figure 7B).

**Figure 8:**
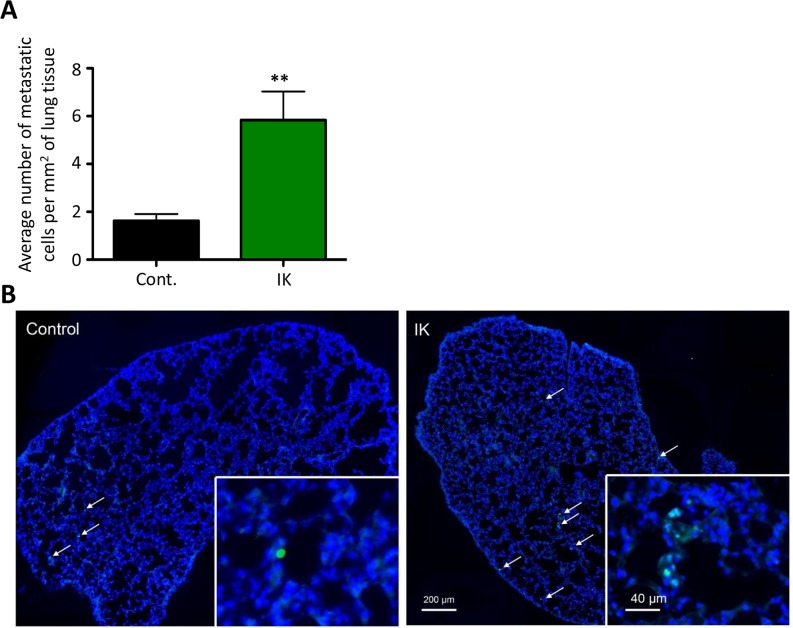
IK over-expression increases metastasis to the lung **(A)** Quantification of HNA positive cells in lung tissue sections collected 4 weeks after mice were injected in the mammary fat pad with MDA-MB-231 cells. Data are mean with SEM of 3 sections per mouse control n =8, IK n = 9 p < 0.01, 2-sample t-test). **(B)** Representative image of HNA staining (green) with DAPI (blue) counterstain in full lung section with arrows indicating positive cells.

We also assessed metastatic dissemination by comparing the number of cells metastasizing to the lung. Lungs were sectioned and stained with anti-HNA antibody and the number of metastasizing cells was quantified. There was a significant increase in the number of metastasizing cells/mm^2^ in MDA-MB-231-IK injected mice as compared to control (p < 0.01, 2-sample t-test; Figure 8A–8B). In summary, IK over-expression significantly increased primary tumor growth as well as dissemination of cells to lung tissue.

## DISCUSSION

Despite decades of research, metastatic cancer continues to have high rates of recurrence and patient mortality. A better understanding of the molecular pathways that promote aggressive behaviors is needed in order to develop treatments aimed at preventing metastasis. IK channels play important functions in proliferation, migration, and invasion as shown by a decrease in these behaviors *in vitro* and *in vivo* with IK inhibition or knock-down [11, 14, 27, 28]. In this study we investigated whether IK was also sufficient to promote these behaviors, as sufficiency would suggest IK plays important roles in upstream signaling pathways driving disease progression. We found that over-expression of IK was sufficient to increase primary tumor growth and metastasis but that it had cell line dependent differential effects on aggressive behaviors *in vitro*. This study adds to data from inhibition studies suggesting differences in behavioral response to potassium channel activation between cell lines of different tumor aggressiveness [15, 29]. Furthermore, there was no significant change in MDA-MB-231 proliferation in response to IK activation, which is in contrast to the previously reported increased proliferation of prostate cancer cell lines [12, 17]. These results suggest important differences exist in the signaling networks downstream of IK activation between different cell lines and support the investigation of primary tissue and tumors to determine if these differences correlate with disease state.

In support of the initial hypothesis that increased IK activity promotes cancer progression, *in vivo* primary tumor growth and metastasis of MDA-MB-231 to the lung was increased by IK over-expression. MDA-MB-231 control and MDA-MB-231-IK cells developed similarly aggressive primary tumors with no observable difference in local tissue invasion or vascularity. This may be because MDA-MB-231 cells are endogenously highly invasive and it may be difficult to observe potentiation in primary tumors. The *in vivo* data are in contrast to the effect of IK over-expression on *in vitro* proliferation and aggressive behaviors of MDA-MB-231 cells. While there was no significant effect of IK on proliferation, migration, or invasion, it is interesting to note that there was a trend of MDA-MB-231-IK having the highest value in each assay. It is possible that IK over-expression results in a small increase of each behavior that is challenging to detect within the inherent variability of the assays. But *in vivo* where the effect of all of these behaviors is combined during tumor growth, the overall effect is amplified and becomes statistically significant. Alternatively, we speculate the opposing *in vitro* and *in vivo* results could reflect a requirement for signaling from the surrounding tumor microenvironment. It is well known that many *in vivo* factors with important effects on cancer cell behavior are not recapitulated *in vitro* and often treatments that reduce aggressive *in vitro* behaviors have no effect *in vivo* [30–32]. Microenvironmental factors that influence IK activation and signaling have not been studied extensively and may have important roles. For example, growth-promoting effects of IK signaling may require highly specialized spatio-temporal activation that is not recapitulated *in vitro*. IK signaling may be initiated by interactions with stromal cell types, activation of specific adhesion molecules, or microenvironmental factors such as hypoxia or inflammatory cytokines, as potassium conductance has been shown to be sensitive to all of these factors [33, 34]. As a specific example, signaling from the human eag-related gene 1 potassium channel (hERG1) is dependent on interactions with integrin receptors, demonstrating one mechanism by which potassium channel activity is linked to a feature of the surrounding microenvironment [35, 36]. Further experiments are needed to determine how IK activity is responsive to microenvironmental factors.

Migration of both MCF-10A and MDA-MB-231 cells was unaffected by IK over-expression and activation suggesting IK activity is insufficient to drive migration. Prior studies have reported that IK inhibition decreases migration in MDA-MB-231 and have supported a role for activation of IK specifically on the lagging edge [13, 18]. Activation lacking spatial regulation, such as occurs with addition of agonist, may have counteracting effects. The effect of increased IK expression on spatially restricted activation is not known. Activation of IK has been reported to increase intracellular calcium [17], allowing for the possibility of a positive feed-back loop whereby activation of some channels increases intracellular calcium which then activates additional channels. High expression of IK could increase positive feedback mechanisms and cause spatially restricted signals to be propagated to larger portions or the entire cell.

IK expression and activation decreased *in vitro* invasion of MCF-10A but not MDA-MB-231 cells. This effect was most pronounced in IK over-expressing cells treated with agonist and thus the degree to which MCF-10A invasion was inhibited correlated with the level of IK activity. Given that there was no change in MCF-10A migration, the decreased invasion is likely related to either an inability to degrade the ECM or dysregulation of volume control preventing volume reduction required to pass through the pores in the membrane. Further studies are needed to distinguish these two possibilities as well as to understand why MCF-10A but not MDA-MB-231 invasion is sensitive to increased IK currents. Surprisingly, IK expression and activation decreased MDA-MB-231 colony formation. Further studies are needed to delineate a mechanism for this result.

In contrast to increased proliferation seen in prostate cancer cells, neither IK over-expression nor activation with 1-EBIO caused a change in MDA-MB-231 proliferation, suggesting the mechanism downstream of IK activation may not be conserved across cancer types [12]. Additionally, proliferation of the non-tumorigenic cell line MCF-10A was decreased by IK activation in both control and IK over-expressing cells but not by IK over-expression alone. There was no change in apoptosis or cell death suggesting the diminished cell number was due to a decrease in the rate of cell division. MCF-10A control and IK over-expressing cells treated with 1-EBIO had a significant increase in the percentage of cells in G2 while IK over-expression alone had no effect on cell cycle distribution. MDA-MB-231, which had no change in proliferation across any condition, also had no significant difference in the percentage of cells in G2. Thus, the accumulation of MCF-10A cells in G2 mirrored the decrease in proliferation, suggesting constant IK activation decreases proliferation of MCF-10A by inhibiting progression from G2 to mitosis. Changes in potassium channel activity and V_mem_ are associated with progression through check points of the cell cycle with depolarization and a decrease in potassium channel activity typically occuring during G2 [5]. Activation of IK may prevent depolarization during late G2 and progression to mitosis. Further investigation is needed to determine if G2 accumulation is due to hyperpolarization specifically, as this would for the first time indicate a requirement for depolarization as part of the G2 checkpoint. The different response seen between MDA-MB-231 and MCF-10A cells suggests the cell lines may have distinct bioelectric profiles with altered sensitivity to bioelectric events. This may be a result of differences in expression of bioelectric sensors including proteins related to calcium signaling, or a difference in the ability to compensate for the increased potassium current. Understanding the bioelectric requirements of the G2 checkpoint may help elucidate novel therapies designed to utilize differences in sensitivity to target specific cell populations.

One possible mechanism to explain the contrasting behavioral response between MCF-10A and MDA-MB-231 is a difference in expression of IK binding partners and other closely associated proteins that differ between the two cell types. IK signaling is dependent on interactions with beta-1-integrin and TRP member calcium channels in other cell types [17, 37]. Little is known about variability of associated proteins and how it affects behavioral response. There are many members within the TRP calcium channel family each with unique activation properties and thus IK associated with different members may have a different effect on calcium dynamics and downstream signaling. While some studies have investigated the requirement of associated proteins to IK signaling, none have investigated the effect of substituting related family members to gauge the response to a more subtle perturbation of the system. Additionally, a recent study found anomalous downstream responses to different IK inhibitors likely caused by maintenance of high intracellular calcium in response to one inhibitor but not the other [38]. Together with our work, these findings suggest the downstream response to IK modulation is complex and point to mechanisms that may make the response cell-type specific. Analysis of additional cell lines and primary cells is needed to determine if the divergent behavioral responses to IK stimulation are characteristic of specific cell types or disease states and to elucidate the mechanism driving the different behaviors.

Prior studies have placed the cancer-associated ion channel IK within a growing number of channels that are required to support aggressive tumor behaviors. This study is the first to demonstrate that IK is also sufficient to promote *in vivo* cancer growth and metastasis. Our findings suggest that IK has a greater impact on signaling pathways driving cancer progression, likely calcium-sensitive pathways, than previously thought. Ion channels are an intriguing potential mechanism of metastasis initiation as they are highly sensitive to microenvironmental factors and therefore could explain why only a small percentage of syngeneic cells go on to form metastases. The results of this study support the ability of IK to increase aggression of already transformed cancer cells. The ability of IK to promote tumor growth and metastasis *in vivo* points to the potential of IK-inhibiting drugs to decrease cancer progression. Furthermore, the opposing behavioral response between cancerous MDA-MB-231 and non-tumorigenic MCF-10A cell lines warrants further study to determine if there are unique mechanisms conferring different sensitivities that could be utilized to formulate targeted therapies.

## MATERIALS AND METHODS

### Generation of pMIG expression plasmid

The retroviral expression plasmid pMIG and a plasmid containing tagRFP (RFP- red fluorescent protein) were purchased from Addgene (#12282 and #37537). A plasmid containing the IK complete coding sequence was purchased from Genecopoeia (GC-0G00902). The IK coding sequence in tandem with P2A and tagRFP was inserted into pMIG to replace the IRES and green fluorescent protein (GFP) sequence using the Gibson cloning method following manufacturer instructions (NEB). Briefly, primers were designed to amplify pMIG excluding the IRES-GFP sequence (forward primer 5′ GCCAAGCTTATCGATAAAATAAAAGA, reverse primer 5′ AATTCCGGCGCCTAGAGA). Primers for the channels and tagRFP were designed with 15-40 bp overhanging sequences to match the adjacent DNA sequence of the final plasmid and to insert the P2A sequence (IK forward primer 5′ CTAGGCGCCGGAATTACCATGGGCGGGGATCTGG, reverse primer 5′ TCTCCTGCTTGCTTTAACAGAGAGAAGTTCGTGGCTCCGGATCCCTTGGACTGCTGGCTGGG; tagRFP forward primer 5′ TCTCTCTGTTAAAGCAAGCAGGAGACGTGGAAGAAAACCCCGGTCCTGTGTCTAAGGGCGAA GAGCTGA, reverse primer 5′ CCTACAGGTGGGGTCTCACTTGTACAGCTCGTCCATGCC). All fragments were amplified by PCR and gel purified. A Gibson reaction was performed using three DNA fragments, the pMIG backbone, channel, and tagRFP, to create the final plasmid (pMIG-IK). A plasmid with only tagRFP inserted was generated as a control (pMIG-RFP).

### Retrovirus production and transduction

Two days prior to transfection, 2 × 10^6^ 293 Plat GP cells (Cell Biolabs) were plated on a 10 cm dish. Transfection was performed with 60 μL of lipofectamine 2000, 6 μg of VSV packaging plasmid, and 12 μg of pMIG-Channel-P2A-RFP plasmid per manufacturer instructions. Cells were incubated in the DNA:liposome complex overnight in a final volume of 6 mL. At 72 hours post transfection, virus media was collected and filtered through a 45 μm filter. Human breast epithelial cell lines were transduced by incubating 50% confluent cells with virus in media supplemented with 6 μg/mL polybrene for 24 hours. At 72 hours post-transduction, retrovirus treated cells were placed in media containing 50 μg/mL G418 for selection. Retrovirus infected cells were expanded for later FACS sorting.

### Cell cultivation

MCF-10A and MDA-MB-231 cell line were purchased directly from ATCC which certified the identity of the cell lines by STR analysis and also certified the cell lines as pathogen-free. Cells were maintained following ATCC recommendations.

### IK activation

Cells were treated with 200 μM 1-EBIO diluted from a 400 mM 1-EBIO stock solution in dimethyl sulfoxide (DMSO) resulting in a final concentration of 0.05% DMSO in the cell medium. Control samples were treated with an equivalent concentration of DMSO as vehicle control.

### Electrophysiology

Patch clamp recordings were performed in the cell attached perforated patch configuration and data were recorded with an Axon DigiData 1550 (Axon Instruments) data acquisition system and an Axopatch 200B (Molecular Devices) amplifier. Data were low pass filtered at 5 kHz, sampled at 50 kHz, and analyzed using pClamp 10 software (Axon Instruments). Patch pipettes were pulled from thin-wall borosilicate glass with a P-97 micropipette puller (Sutter Instruments) and fire polished resulting in 4-7 MΩ resistance pipettes. Pipettes were filled with intracellular electrode solution (5 mM NaCl, 145 mM KCl, 2 mM MgCl_2_, 1 mM CaCl_2_, 1.57 mM ethylene glycol-bis(2-aminoethylether)-*N-N-N’-N’*-tetraacetic acid (EGTA), 10 mM 4-(2-Hydroxyethyl)piperazine-1-ethanesulfonic acid (HEPES), pH adjusted to 7.4 with 1 M KOH) supplemented with 150 ng/ml Nystatin to induce pore formation. Prior to recording, cells plated on glass coverslips were perfused with external bath solution (144 mM NaCl, 5.4 mM KCl, 1 mM MgCl_2_, 2.5 mM CaCl_2_, 5.6 mM Glucose, 5mM HEPES, adjusted to pH 7.2 with 1 M NaOH). After initial seal formation, with a minimal seal resistance cut off of 1.0 GΩ, perforation was assayed by monitoring the capacitive current transient to a 2.5 mV step with -30 mV holding potential. Recordings were acquired once the series resistance was below 75 MΩ. V_mem_ recordings were taken for 30 seconds in control extracellular solution supplemented with vehicle control with no fluid-flow, during 30 seconds of bath exchange with the same solution, during 30 seconds of bath exchange with bath solution containing 200 uM 1-EBIO, and for 30 seconds in 200 μM 1-EBIO with no fluid flow. The reported V_mem_ values are the average from the last 20 seconds recorded in static fluid from each condition. For the current-voltage protocol, cells were held at -30 mV followed by 50 ms test pulses between -120 mV and 80 mV in 40 mV steps. Current density was calculated by dividing the average current during the voltage step without leak subtraction by the whole cell capacitance.

### RNA isolation, purification, and quantitative PCR

RNA was isolated from confluent cells in a 6-well plate. Cells were rinsed with PBS and 200 μL RNA-later (Ambion) was added before scraping and transferring cells to a 1.5 mL tube. RNA was purified using an RNeasy mini kit (Qiagen) per manufacturer instructions. The concentration of RNA was determined with a NanoDrop 2000 (Thermo Scientific). Reverse transcription reactions were performed with 1 μg of RNA in a 20 μl reaction using iScript Reverse Transcription Supermix (Bio-Rad) following manufacturer instructions. Transcript expression levels were quantified with a Bio-Rad CFX96 Real Time System. Reactions were performed in 20 μL using iQ SYBR Green Supermix (Bio-Rad) and 40 ng cDNA with the following reaction conditions: 95°C 10 min, 40 cycles of 95°C 30 sec, 58°C 1 minute, 72 C° 1 minute. The primers used to amplify an IK fragment were forward primer 5′ CTGCTGCGTCTCTACCTGG and reverse primer 5′ AGGGTGCGTGTTCATGTAAAG and GAPDH was used as the housekeeping gene with forward primer 5′ TTCGACAGTCAGCCGCATCTTCTT and reverse primer 5′ ACCAAATCCGTTGACTCCGACCTT. To detect SK and compare to IK expression levels, QPCR was performed using Taqman probes (Thermo Scientific) for SK, IK, and GAPDH with Taqman universal master mix II (Thermo Scientific) following manufacturer's instructions.

### Proliferation assay

MCF-10A cells were plated at 7.5 × 10^3^ cells/cm^2^ and MDA-MB-231 cells were plated at 1 × 10^4^ cells/cm^2^ in 6 well plates. Cells were allowed to attach in normal medium for 3 h followed by exchange to media supplemented with drug treatment. Cells were trypsinized and counted with a TC 10 automated cell counter (BioRad) daily for 4 days with a media change after 2 days.

### Apoptosis, cell death, and cell cycle analysis

Cells were plated at 5 × 10^4^ cells/cm^2^ and allowed to attach for 3 hours. Medium was exchanged to medium supplemented with vehicle or 200 μM 1-EBIO and cells were incubated for 24 h. To quantify the percentage of apoptotic and dead cells, cells were trypsinized, washed in PBS, and incubated in 0.1% Live/Dead Fixable Violet Dead Cell stain for 30 min. Cells were washed in Annexin V Binding Buffer and incubated in Annexin V conjugated to FITC diluted 1:125 in Annexin V Binding Buffer (Invitrogen). For cell cycle analysis, cells were fixed in 70% ethanol and washed in phosphate-buffered saline (PBS). Cells were incubated in 50 ng/mL propidium iodide (Invitrogen), 250 ng/mL RNase diluted in PBS for 1hr. Cell fluorescence was detected using an LSR II (Becton Dickenson) fluorescent cell analyzer and data were analyzed with FlowJo software.

### Migration assay

MDA-MB-231 cells were plated at 2.5 × 10^5^ cells/cm^2^ in a 24 well plate and were serum starved in 1% FBS media overnight. A scratch was made with a yellow tip and medium was exchanged with low serum medium containing drug treatment. Phase contrast images were acquired of the initial scratch. 10 h post scratch, cells were incubated in 0.5 μg/mL calcein AM for 15 minutes and fluorescent images were acquired. ImageJ was used to calculate wound healing by first manually outlining the wound at time 0 hr to create a region of interest (ROI). The fluorescent 10 h image of calcein AM stained cells was converted to a binary image such that white pixels corresponded to the surface area covered by cells. The percentage of white pixels within the initial wound ROI was quantified to measure wound healing. Scratch assays with MF-10A cells were performed similarly but 24-well plates were seeded with 3.75 × 10^5^ cells/cm^2^ cells, low serum medium consisted of normal MCF-10A medium with 0.5% horse serum and 2 ng/mL EGF, and bright field images were acquired 0 h and 12 h after scratching and the area of the scratch wound was measured with image J.

### Invasion assay

The day prior to invasion assays, cells were placed in low serum medium. Basement membrane coated transwells with 6.5 mm diameter and 8 μm pore size (BD Biosciences) were pre-incubated with 500 μL DMEM in the upper chamber for 2 hours. MDA-MB-231 cells were diluted to 2.5 × 10^5^ cells/mL and MCF-10A cells were diluted to 5 × 10^5^ cells/mL in low serum media and 200 μL of the cell suspension was placed in the upper chamber giving 5 × 10^4^ and 1 × 10^5^ cells per well respectively. The bottom chamber was filled with 600 μL of normal full serum media as a chemoattractant. After 16 h for MDA-MB-231 cells or 24 h for MCF-10A cells, transwells were rinsed in PBS and cells were scraped off of the upper chamber. Remaining cells were fixed in 4% PFA for 10 minutes. Transwells were rinsed in PBS and stained with 1 μg/mL 4′, 6-Diamidino-2-Phenylindole Dihydrochloride (DAPI) for 10 minutes. The number of invading cells was counted in 4 fields of view for each sample.

### Colony formation assay

MCF-10A cells were plated at 3 × 10^4^ cells per well and MDA-MB-231 were plated at 5 × 10^3^ cells per well of a six well plate. The wells were first coated in 1.5 mL of 0.8% agarose diluted in normal culture media. Cells were plated in 1.5 mL of 0.4% agarose diluted in normal culture media and the agarose was allowed to set at room temperature for 1 hr. Samples were cultured for 4 weeks with 0.5 mL culture media added on top of the culture media with media exchanges every 2-3 days. After 4 weeks, samples were fixed in 10% formalin for 30 minutes and incubated in .005% crystal violet for 1 hr. Samples were rinsed in water until the washes were clear of stain. Images were acquired using a ChemiDoc XRS+ gel imager (BioRad) and associated software. Colonies were counted using ImageJ.

### Orthotopic breast tumor model

All procedures involving animals were approved by the University of York Ethical Review Process and under the authority of a UK Home Office project License. Six week old female Rag2^−/−^, Il2rg-^/−^ mice (Yorkshire Cancer Research Unit, University of York) were selected at random for MDA-MB-231 control or MDA-MB-231-IK injection. A 5 × 10^5^ cell suspension was prepared in 20% v/v matrigel in saline and injected into the left inguinal mammary fat pad of isoflurane anaesthetized mice. A total of 14 and 11 mice were injected with MDA-MB-231 control and MDA-MB-231-IK cells respectively across 4 independent experiments. Tumors did not take in 3 of the control and 2 of the IK expressing mice and were not included in analysis giving n = 11 for MDA-MB-231 control and n = 9 for MDA-MB-231-IK. The length and width of primary tumors was measured daily with calipers and the tumor volume was calculated as 0.5 × (length × width^2^). Mice were euthanized 28 days after injection and tumors and lungs were fixed in 4% paraformaldehyde and frozen.

### Immunohistochemistry

H&E staining and immunohistochemistry were performed as described [39]. The following primary antibodies were used: mouse anti-IK (1:20; Alomone); rabbit anti-Ki67 (1:5000; Abcam); rabbit anti-activated caspase-3 (1:200; R&D Systems); rabbit anti-CD31 (Santa Cruz Biotechnology); mouse anti-HNA (1:100; Millipore). Secondary antibodies were Alexa-488-conjugated goat anti mouse/rabbit (1:500; Invitrogen). Samples were mounted in Prolong Gold with DAPI (Invitrogen). Sections were scanned at 20X using a Zeiss AxioScan.Z1 slide scanner. Images were exported into ImageJ for processing. Brightness/contrast was adjusted using the ImageJ “Auto” function. Density of Ki67^+^ or activated caspase-3^+^ cells, CD31^+^ vessel structures, and metastasis to lungs were measured across scanned images of whole sections, blinded to treatment [39, 40].

### Statistics

Statistics were analyzed in Prism 5 and significance was determined using a two sample t-test or a one-way ANOVA followed by either a Dunett post test (to compare multiple conditions to a single control) or a Tukey post test (for a comparison of all conditions), alpha = 0.05. In cases where a comparison is made between two specific conditions from within an ANOVA the q value is given, otherwise only the p-value is given. All results are presented as mean with standard deviation from 3 independent experiments unless otherwise noted.

## SUPPLEMENTARY MATERIALS FIGURES



## Abbreviations

1-EBIO: 1- Ethylbenzimidazolinone
DAPI: 4′, 6-Diamidino-2-Phenylindole Dihydrochloride
DMSO: dimethyl sulfoxide
EGTA: ethylene glycol-bis(2-aminoethylether)-*N-N-N′-N′*-tetraacetic acid
FACS: fluorescence activated cell sorting
FBS: fetal bovine serum
HEPES: 4-(2-Hydroxyethyl)piperazine-1-ethanesulfonic acid
IK: intermediate conductance calcium-activated potassium channel
PBS: phosphate-buffered saline
RFP: red fluorescent protein
ROI: region of interest
SK: small conductance calcium-activated potassium channel
V_mem_: membrane potential
