# Supplementary material for: IK channel activation increases tumor growth and induces differential behavioral responses in two breast epithelial cell lines
Source: Oncotarget. 2017 Mar 21;8(26):42382–97. doi: 10.18632/oncotarget.16389 (PMC5522074; doi:10.18632/oncotarget.16389)
Supplement: Supplementary file 1 [file oncotarget-08-42382-s001.pdf]

IK channel activation increases tumor growth and induces differential behavioral responses in two breast epithelial cell lines

SUPPLEMENTARY FIGURES

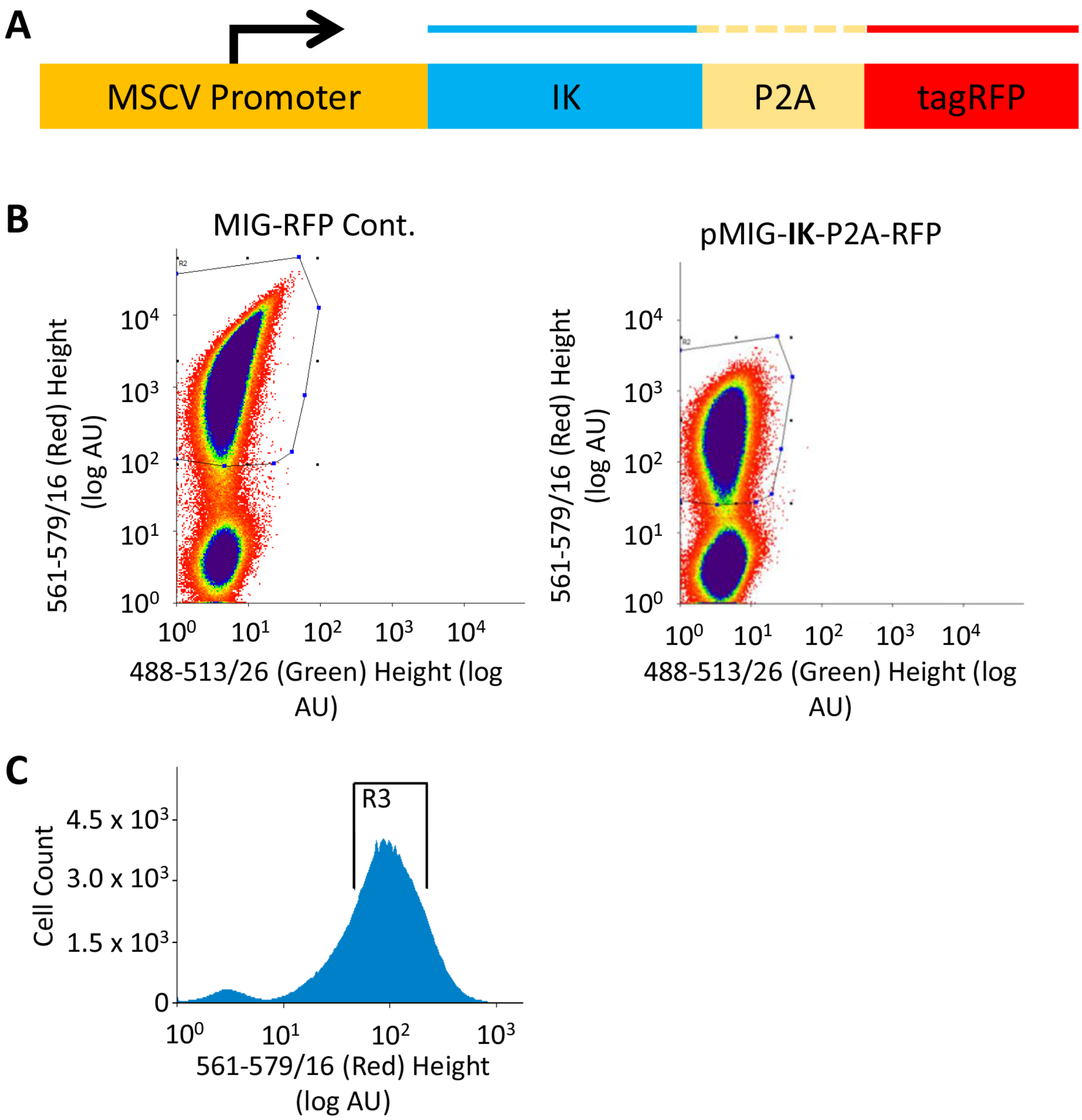

Supplementary Figure 1: IK expression strategy and selection of RFP positive cells. **(A)** Schematic of IK expression plasmid. IK was inserted in tandem with the P2A peptide bond skip sequence and RFP causing translation to produce a separate IK and RFP protein. Expression was driven by the MSCV promoter giving high constitutive expression. A control plasmid was also created that lacked the IK sequence. **(B)** Plots of FACS sort of infected MDA-MB-231 cells. Cells infected with pMIG-RFP control vector (left) and pMIG-IK (middle) sorted based on RFP fluorescence and cells within the polygon were selected. **(C)** In order to create a more homogenously expressing population, the sorted MDA-MB-231-IKpopulation was expanded and sorted a second time selecting for a narrower range of highly expressing cells (cells within R3 selected).

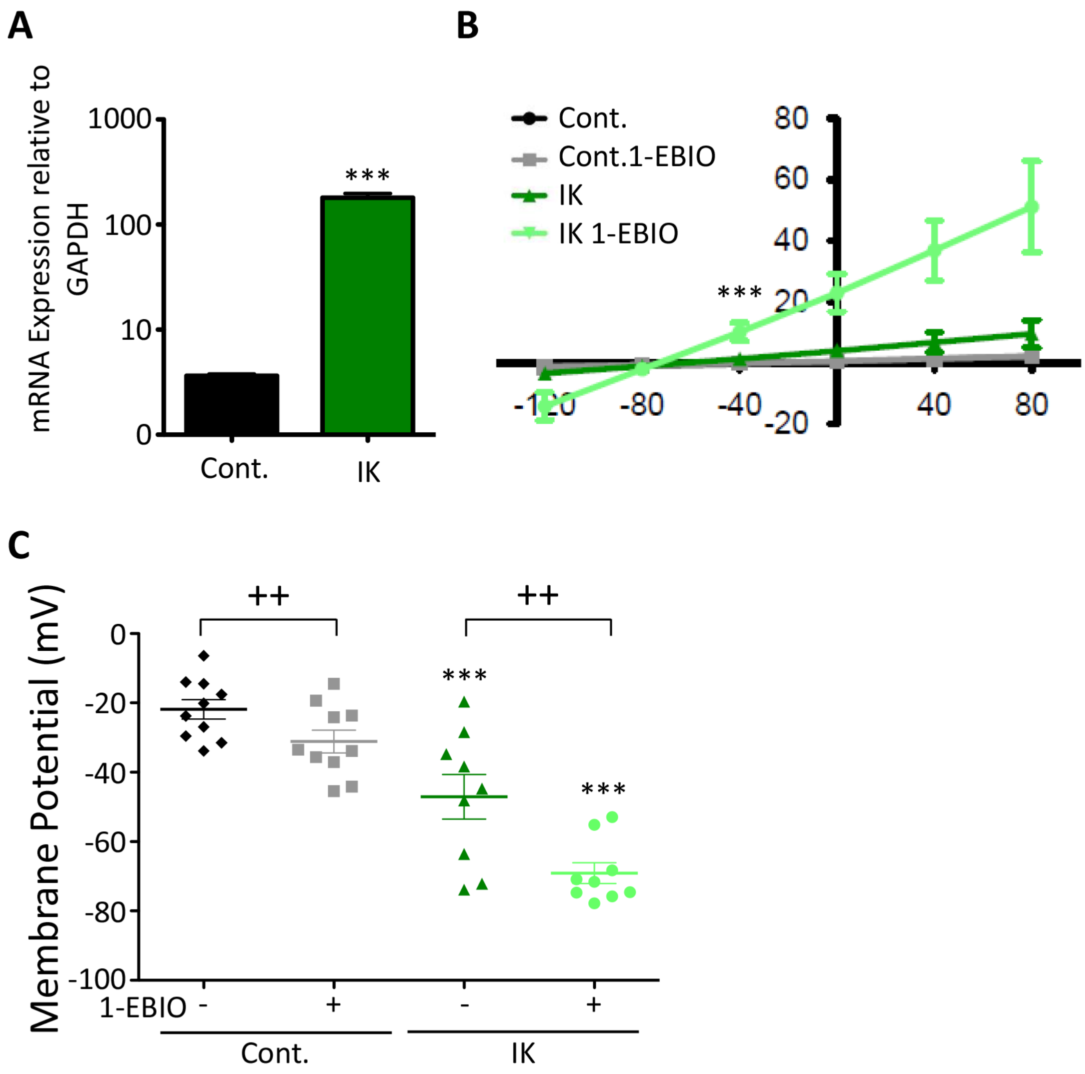

Supplementary Figure 2: Functional contribution of IK over-expression to MCF-10A current density and V_{mem}. (A) IK mRNA expression levels relative to GAPDH in total RNA collected from MCF-10A infected with pMIG-RFP (Cont.) or pMIG-IK (IK) and selected for RFP fluorescence by FACS. Data are presented as mean with SEM of 3 independent replicates, (*** $p < 0.01$, 2 sample t-test). **(B)** Endogenous and 1-EBIO induced current-voltage relationship in control and IK-expressing cells recorded in the cell attached perforated patch configuration from MCF-10A cells. Data are presented as mean with SEM from a minimum of 7 cell recordings. The current density was significantly increased in MCF-10A-IK 1-EBIO treated cells compared to control vehicle treated, control 1-EBIO treated, and MCF-10A-IK vehicle treated cells (*** $p < 0.001$, 1-way ANOVA of -40 mV current density) **(C)** V_{mem} averaged over 20 seconds from recordings of same cells as B. Data points represent individual cells, bars show mean with SEM (*** $p < 0.001$, 1-way ANOVA significantly different than control vehicle treated; ++ $p < 0.01$, paired t-test of same cell before and after 1-EBIO treatment).

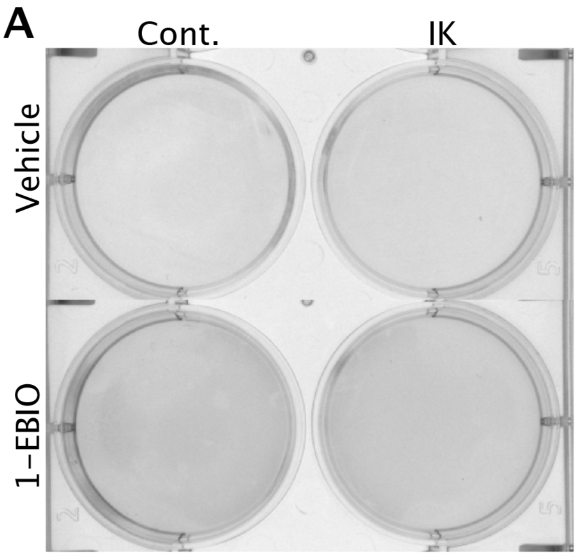

Supplementary Figure 3: Neither IK over-expression or activation are sufficient to induce MCF-10A colony formation in soft agarose. (A) Bright field images of crystal violet stained MCF-10A control and MCF-10A-IK cells grown in soft agarose and treated with vehicle control or 1-EBIO for 28 days. There was no colony formation in any condition.
